# District division administrative disaggregation data framework for monitoring leaving no one behind in the National Health Insurance Fund of Sudan: achieving sustainable development goals in 2030

**DOI:** 10.1186/s12939-020-01338-6

**Published:** 2021-01-06

**Authors:** Ashraf Mansour, Nithat Sirichotiratana, Chukiat Viwatwongkasem, Mahmud Khan, Samrit Srithamrongsawat

**Affiliations:** 1 Department of Public Health Administration, Faculty of Public Health, Mahidol University, Bangkok, 10400 Thailand; 2 Department of Biostatistics, Faculty of Public Health, Mahidol University, Bangkok, Thailand; 3 Arnold School of Public Health, University of South Carolina, Columbia, USA; 4 Faculty of Public Health Ramathibodi Hospital, Mahidol University, Bangkok, Thailand

**Keywords:** District division administrative disaggregation data, Leaving no one behind, Sustainable development goals

## Abstract

**Background:**

The aim of this study is to monitor the concept of ‘leaving no one behind’ in the Sustainable Development Goals (SDGs) to track the implications of the mobilization of health care resources by the National Health Insurance Fund (NHIF) of Sudan.

**Methods:**

A cross-sectional study was used to monitor ‘leaving no one behind’ in NHIF by analyzing the secondary data of the information system for the year 2016. The study categorized the catchment areas of health care centers (HCCS) according to district administrative divisions, which are neighborhood, subdistrict, district, and zero. The District Division Administrative Disaggregation Data (DDADD) framework was developed and investigated with the use of descriptive statistics, maps of Sudan, the Mann-Whitney test, the Kruskal-Wallis test and health equity catchment indicators. SPSS ver. 18 and EndNote X8 were also used.

**Results:**

The findings show that the NHIF has mobilized HCCs according to coverage of the insured population. This mobilization protected the insured poor in high-coverage insured population districts and left those living in very low-coverage districts behind. The Mann-Whitney test presented a significant median difference in the utilization rate between catchment areas (*P* value < 0.001). The results showed that the utilization rate of the insured poor who accessed health care centers by neighborhood was higher than that of the insured poor who accessed by more than neighborhood in each state. The Kruskal-Wallis test of the cost of health care services per capita in each catchment area showed a difference (*P* value < 0.001) in the median between neighborhoods. The cost of health care services in low-coverage insured population districts was higher than that in high-coverage insured population districts.

**Conclusion:**

The DDADD framework identified the inequitable distribution of health care services in low-density population districts leaves insured poor behind. Policymakers should restructure the equation of health insurance schemes based on equity and probability of illness, to distribute health care services according to needs and equity, and to remobilize resources towards districts left behind.

## Background

The greatest challenge faced by the world today is poverty eradication [1]. Universal health care (UHC) was proposed as a financial system to protect poor people from financial hardship [2]. UHC implemented in Sudan through the National Health Insurance Fund (NHIF) sought to reduce inequalities in health outcomes to protect poor populations [3].

Globally, 56% of the population in rural areas suffers from a lack of health care service coverage, compared to only 22% in urban areas [4]. The situation is most severe in Africa, where 77% of the rural population suffers from a lack of health care service coverage compared to 50% of the urban population, who lack access to necessary services due to the shortage of health workers [4]. In Sudan, 74% of health care facilities are unable to provide essential health care services, even though the geographical coverage of HCCs is 86% [3]. These statistics on the coverage of primary health care facilities show an inequity between states with a range of 1 health care center: 3039 persons to 1 health care center: 20,770 persons [5]. The percentage of the population not covered by health care facilities within 5 km differs between states, from 0.1 to 42.3 [5]. A survey from 2009 indicated that an urban dweller was 60% more likely to have utilized outpatient services than a person who lived in a rural area [5]. Resource allocation was not available for the poor; lower-income quartiles received 13% of public expenditures compared to 26% for higher-income quartiles [5]. Poor patients utilized health care almost ten times less frequently than rich patients [5]. As a result, tracking the implications of health resource allocation by the NHIF is crucial for poverty eradication.

The concepts of health equity within SDGs 1, 3.8, 10 and 17.18 were synthesized to explore the functions of the NHIF. The desired outcomes for the poor in SDG 1 are to end all forms of poverty everywhere, to achieve significant health coverage of the poor and vulnerable through the national application of social protection systems and measurements by 2030, to consider that everyone has the equal right of access to basic services, and to accelerate investment in poverty eradication at all levels through the formulation of pro-poor policy frameworks [1]. SDG 3.8 emphasizes the achievement of UHC with financial risk protection [1]. The targets of SDG 10 are to decrease inequality within and between countries, to ensure that everyone has equal opportunity, to eliminate inequalities in outcomes and to formulate social protection polices and policies for achieving equality [1]. SDG target 17.18 focuses on providing meaningful high-quality data with reliability [1]. It suggests the disaggregation of data according to geographical location, gender, age, income, race, ethnicity, disability, migratory status, and other features applicable in countrywide contexts [1].

As a result, SDG goals 1 and 10, targets 3.8 and 17.18, address the concept of ‘leaving no one behind’ to accomplish health equity for all people everywhere to contribute to poverty eradication. The NHIF becomes a social protection mechanism, and its function is to remobilize health care services to decrease inequity in accessibility and utilization and to consider everyone in data analysis.

Innovations are needed to apply the concept of ‘leaving no one behind’ to fill the knowledge gap that monitors health equity, according to WHO studies reports and World Health Statistic 2017 monitoring health for SDGs. Innovations in disaggregation of data are needed to implement the concept of ‘leaving no one behind’ in NHIF information systems [6–8]. Innovation is needed to monitor health equity within disadvantaged groups to determine who is being left behind in health equity [6], to monitor the distribution of health care resources between areas to determine where disadvantaged groups are being left behind [6], to measure health equity by two dimensions and to connect the underlying cause to health equity output to understand why disadvantaged subgroups are being left behind [6].

Up to 2015, the process of the NHIF information system provided information concerning the distribution of the insured population by states and sectors, the distribution of health care centers by districts and states, the utilization of health care services by sectors, district, state, age, sex, health care center, type of health care facility, specialist health care services, referral of health care services, types of health care services, types of diagnostic and laboratory services, inpatient health care services, chronic diseases, diseases by system and cost of utilization of health care services [9].

This study analyzed the frameworks of the WHO [10–13], the health care system of Sudan [5], and the NHIF information system [9] to monitor the reduction of health inequity in UHC programs for poverty eradication. These frameworks aggregate the data with regard to age, gender, education, socioeconomic status, income and place of residence by district, state, rural or urban area, migrants and minorities [12, 14, 15]. The frameworks measure the distribution of health care facilities, population coverage, and access to essential health care services by population cover within 5 km and not within 5 km and utilization by place of residence [12], district [15] and state [14, 15].

The frameworks do not take into account how to innovate the new disaggregation of data to track equal opportunity and inequalities of outcomes between populations with the target of detecting the poor left behind. Furthermore, the frameworks do not consider the main function of a UHC program, which is to remobilize resources towards areas left behind by equitable distribution to manage inequitable access to achieve the target of no insured poor left behind.

The goal of developing the DDADD framework in this study was to monitor the concept of leaving no one behind in health equity in SDGs to track the equitable implications of the mobilization of health care resources by NHIF towards UHC. The objectives were to identify who is being left behind, why they are being left behind, where they are being left behind and how to protect them from being left behind.

The unique DDADD framework elaborates the main function of the NHIF, which is to reallocate health care resources towards health inequity reduction within the system. The framework was constructed around the DDA catchment areas of HCCs and by utilizing the concept of ‘leaving no one behind’ in health equity in the SDGs to provide information about the equitable implications of the mobilization of health care resources by the NHIF towards districts left behind.

## Methods

This study synthesized Andersen’s behavioral model of health care utilization with the principles of health equity in the SDGs in the NHIF setting and the district division administrative (DDA) catchment area distribution of HCCs. This synthesis applied the concept of ‘leaving no one behind’ in the WHO’s Framework for Designing Health Information Systems, which consists of data collection, data transmission, data processing, data analysis and information [16]. The concept has four dimensions: who is being left behind, why they are being left behind, where they are being left behind and how to protect them from being left behind [6]. This synthesis innovates the DDADD framework.

Andersen’s behavioral model is commonly used to evaluate factors that are associated with patient utilization of health care services. This model suggests that individual determinants of health care utilization are predisposing, enabling, and need factors [17–20]. Enabling factors consist of an individual’s income, health insurance status, availability of HCC, and access to HCC; the enabling factors were chosen because the majority of the factors can be transformed by policymakers [17–20]. The model was applied to study the influencing enabling factors associated with health service utilization in the NHIF. Patients utilize health care services more when they are located near them than when they are located at a distance from health care services [21].

Sudan is divided into 18 states and 189 districts. The cross-sectional study analyzed the secondary data from the NHIF’s information system for the year 2016. This study included all insured populations who visited health care facilities in a district or a state in addition to all HCCs that provided health care services by general practitioners (GPs) in 2016. Any HCC that had contracted medical assistance from the NHIF’s health care service package was excluded. In this study, the data did not include the rich and poor populations from Khartoum State. The data on income- insured visitors to health care facilities were gathered from 13 states, and data from five states were missing.

### Disaggregation of data

**Fig. 1:**
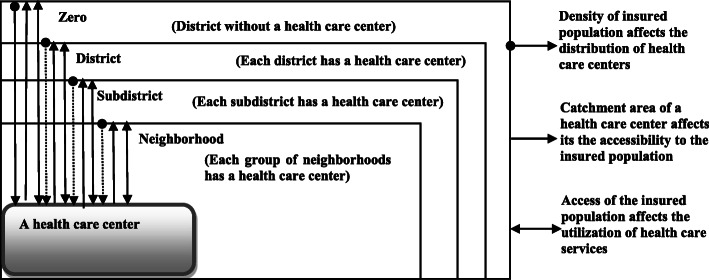
The District Division Administrative Catchment Area Disaggregation of Data Framework

The DDA of Sudan is composed of neighborhoods, subdistricts and districts [22]. The health map of the NHIF targets the insured population of 10,000 in a 5 km square covered by HCC with a GP as its provider [23]. This implies the health equity in the SDGs in the NHIF setting, in which every insured person has access to HCC by neighborhood catchment area and the utilization of health care services is equitable between the insured poor and the insured rich and within the insured poor. The study found that the catchment areas of HCCs were neighborhood, subdistrict and district based on the distribution of HCCs [22] (Fig. 1). If an HCC covered a group of neighborhoods, the catchment area was the neighborhood; if there was an HCC in a subdistrict, the catchment area was subdistrict; and if there was an HCC in a district, the catchment area was the district. When there was no HCC, the catchment area was zero (Fig. 1).

### Study variables

The dependent variables used in this analysis were the catchment area distribution of HCCs, the catchment area coverage of districts, DDA insured accessibility, the insured catchment area utilization of health care services, the income-insured catchment area utilization of health care services and the catchment area cost of health care services. The independent variables were the density of the insured population, the catchment area distribution of HCCs, DDA insured accessibility and DDA income-insured accessibility.

This study measured the catchment area distribution of HCCs by the number of HCCs available according to the DDA catchment areas. The catchment area coverage of districts was accounted for by the number of districts based on DDA catchment area coverage. DDA insured and income-insured accessibility was accounted for by the number of insured, the insured poor and the insured rich coverage in the DDA catchment area coverage. The insured catchment area utilization of health care services was measured by insured visitors to health care facilities by DDA catchment areas. The income-insured catchment area utilization of health care services was measured by insured poor or insured rich visitors to health care facilities by DDA catchment areas. The density of the insured population was measured by insured coverage in the DDA catchment areas. The DDA catchment area ranges were neighborhood, subdistrict, district, and zero.

The income-insured accessibility and income-insured utilization of health care services variables predict that the insured population is being left behind. The catchment area cost of health care services and the density of the insured population explore why they are being left behind. The catchment area distribution of HCCs and the catchment area district coverage examine where they are being left behind. The outputs of these measurements will provide policymakers with information about how to protect the insured population from being left behind.

### Analytical approach

The goal of developing the DDADD framework in this study was to monitor the concept of leaving no one behind in health equity in SDGs to track the equitable implications of the mobilization of health care resources by the NHIF towards UHC. The objectives were to identify who is being left behind, why they are being left behind, where they are being left behind and how to protect them from being left behind.

The analytic methods of the DDADD framework were developed to explore the effect of the density of the insured population on the distribution of HCCs in the catchment area to identify districts left behind, to discover the effect of the catchment area distribution of HCCs on income-insured accessibility in order to know who is left behind, to track the effect of income-insured catchment area accessibility on the income-insured utilization of health care services in order to know who is left behind in terms of utilization and to determine the effect of the density of the insured population on the cost of health care services in the catchment area in order to formulate financial policy.

The database was measured by count, percentage and maps of Sudan. The unit of analysis was the district. SPSS ver. 18 and EndNote X8 were used. Nonparametric tests were implemented to analyze the data because of the abnormal distribution of the data.

The insured catchment area utilization of health care services was investigated by using the Mann-Whitney test, and ratio itself. The Insured Catchment Area Utilization Rate Indicator was used to explore the difference between the Neighborhood (N) N-catchment areas and the More Than Neighborhood (MTN) MTN-catchment area utilization rate at the state and national levels in comparison with the geographical utilization ratio in the same state. The N- or MTN-catchment area utilization rate was measured by the catchment area utilization ratio. The catchment area utilization ratio was the number of the N- or MTN-catchment areas with insured visitors to health care facilities at the state level divided by the N or MTN accessible to the insured population at the state level.

The Income-insured Catchment Area Utilization Rate Indicator was developed to track the effect of DDA accessibility on the catchment area utilization of the insured poor who had been left behind from UHC. This indicator was created to understand why the insured poor were left behind and how to formulate a solution [6]. The utilization rate indicator was measured by using the ratio of the income-insured catchment area utilization to income-insured DDA accessibility. It was composed of the following: the poor N-catchment area utilization rate, the poor MTN-catchment area utilization rate, the rich N-catchment area utilization rate and the rich MTN-catchment area utilization rate.

The Catchment Areas Cost of Health Care Services Indicator was measured by American dollars (USD) per capita and was analyzed using the Kruskal-Wallis test and ratio. The indicator was calculated by dividing the USD cost of health care services according to catchment areas by the amount of insured DDA accessibility. The Sudanese pound was converted to USD, and the amount was calculated by dividing the total cost of health care facilities by seven, which was the buying rate at the time of the study [24]. Mahidol University and the NHIF gave approval to conduct this study.

## Results

The table of catchment area distribution for the general characteristic indicators shows the effect of the density of the insured population on the catchment area distribution of HCCs and detects whether any insured poor were left behind (Table 1). The data were collected from 18 states. The results showed that the population of Sudan was 37,418,999 in 2016, and the NHIF covered 43.8% of the Sudanese population (Table 1). The trend in the density of the Sudanese population and the insured population declined dramatically from very high density in the N-catchment areas to very low density in the district catchment areas and zero catchment areas (Table 1). The trend of the distribution of HCCs displayed a significant gap between the N- and MTN-catchment area districts. The statistics showed that four-fifths of the total HCCs were distributed in one-fifth of the N-catchment area districts, and 70% of the insured population lived in those districts (Table 1). On the other hand, one-fifth of the total HCCs were mobilized for four-fifths of the MTN-catchment area districts, and 30% of the insured population lived in those districts (Table 1).

**Table 1:** Catchment Area Distribution of General Characteristic Indicator

			Catchment area							
Variables	Neighborhoods		Subdistrict		District		Zero		Total	
	Number	%	Number	%	Number	%	Number	%	Number	%%
**Sudanese Population**	19,240,308	51.42	10,391,005	27.77	6,884,931	18.40	902,755	2.41	37,418,999	100
**Insured population**	11,367,127	69.33	3,320,733	20.25	1,518,913	9.26	176,227	1.07	16,396,484	100
**Poor insured population**	3,054,274	46.77	2,068,346	31.67	1,252,875	19.18	155,003	2.37	6,530,498	100
**Rich insured population**	3,625,538	70.23	1,249,621	24.21	266,038	5.15	21,224	0.41	5,162,421	100
**District**	45	23.81	70	37.04	62	32.80	12	6.35	189	100
**Health care center**	1025	78.07	217	16.53	71	5.41	0	0.00	1313	100

The following measurements monitored the equitable implications of the mobilization of health care resources by the NHIF on the reduction of health inequity to leave no poor behind. The Mann-Whitney test displayed a significant median difference in the utilization rate of HCCs between the N- and MTN-catchment areas (*P* < 0.001). The statistics showed a median of 1.37 and an interquartile range (IQR) of 0.95 in the N-catchment areas, whereas the statistics showed a median of 0.42 and an IQR of 0.63 in the MTN-catchment areas.

**Fig. 2:**
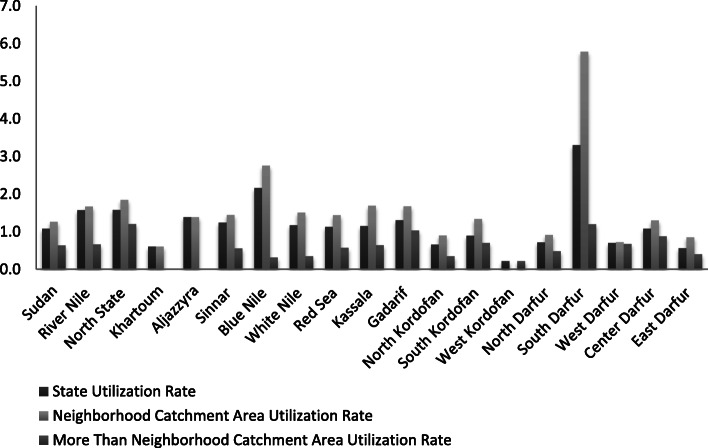
Insured Catchment Area Utilization Rate Indicator Data about the total utilization of health care services were missing in 13 cases

This study compared the geographically insured utilization trend with the utilization rate of the insured in the N-catchment and the MTN-catchment areas at the state level (P < 0.001) and the national level (Fig. 2). The statistics showed that the insured N-catchment area utilization rate was higher than the insured MTN-catchment area utilization rate at the state and national levels, with variation from state to state (Fig. 2). Khartoum and Aljazzyra States had access to HCC by neighborhood, whereas subdistrict accessibility was only found in West Kordofan State (Fig. 2). The national and state geographical utilization rates present a trend of the insured N-catchment area utilization rate compared to the MTN-catchment area utilization rate in each state except Khartoum and West Kordofan States (Fig. 2).

**Fig. 3:**
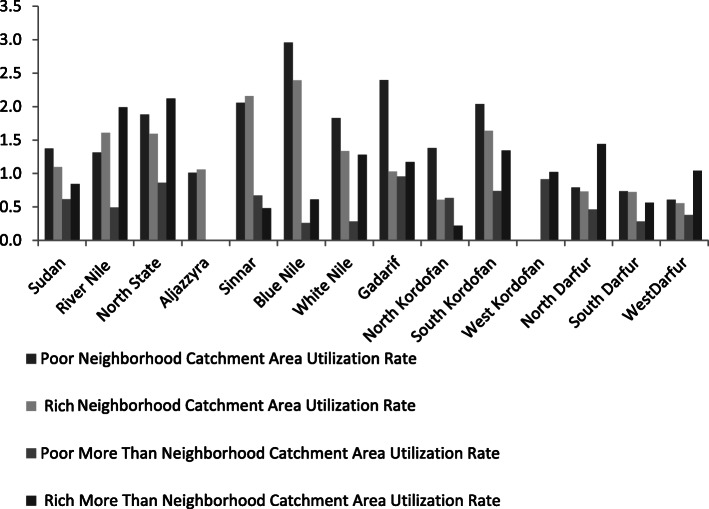
Income Insured Catchment Area Utilization Rate Indicator. Data were received from 13 states of Sudan

The poor N-catchment area utilization rate was found to be higher than the poor MTN-catchment area utilization rate in each state. At the national level, there was variation from state to state (Fig. 3). The majority of states and the national-level poor MTN-catchment area utilization rate presented a lower rate than the rich MTN-catchment area utilization rate, except for North Kordofan and Sinnar States, which displayed variations between the states (Fig. 3). However, the poor N-catchment area utilization rate was higher than the rich N-catchment rate at the state and national levels, except for River Nile State, which was low.

The Kruskal-Wallis test of the catchment area cost of health care services per capita showed a difference (*P* value < 0.001) in the median between neighborhoods of 1.47 IQR 5.67, subdistrict 2.99 IQR 6.98, district 6.29 IQR 4.73, and zero 0.00 IQR 0.00. The catchment area cost of health care services per capita for the NHIF in 2016 differed between catchment areas. The costs of health care services per capita in the neighborhood catchment area, subdistrict catchment area, district catchment area, and zero catchment area were 2.8, 3.5, 4.0, and 3.2 USD, respectively.

**Fig. 4:**
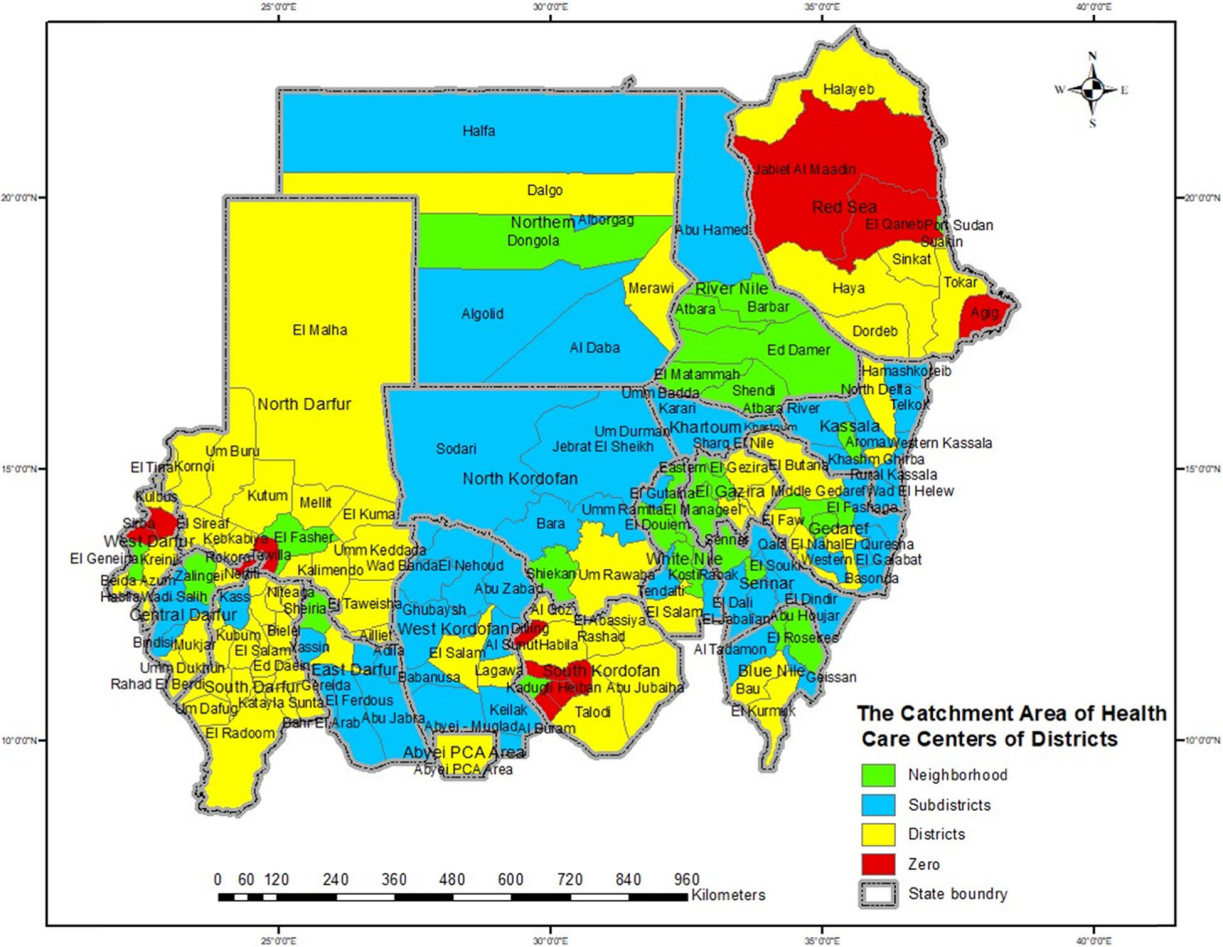
Map of Sudan, the Catchment Area of Health Care Centers of Districts. Source: The software of the map from OCHA

**Fig. 5:**
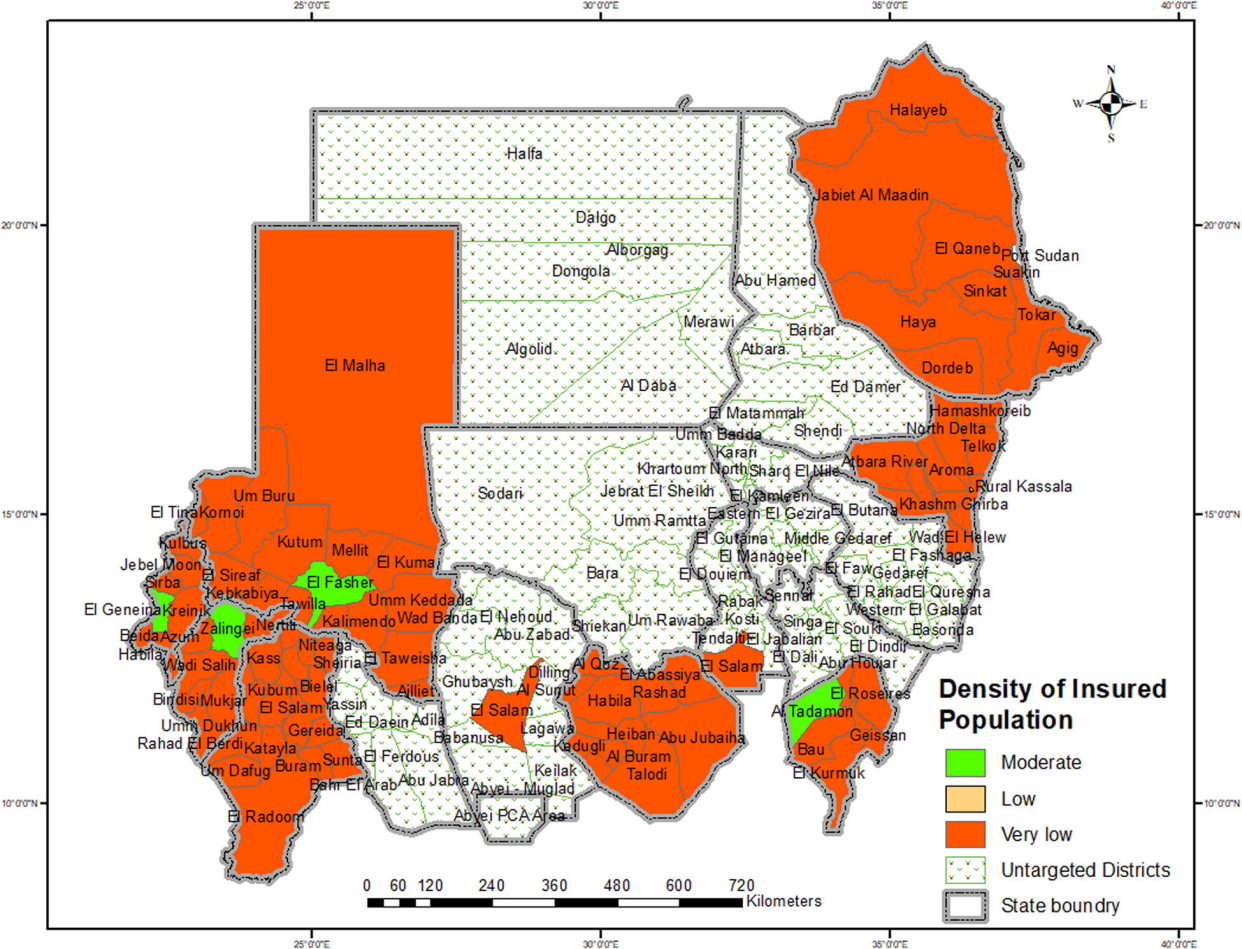
Map of Sudan, the Density of Insured Population of Districts. Source: The software of the map from OCHA

The catchment areas of HCCs of the district map of Sudan indicators showed that district and zero catchment area districts had very low insured population densities with very low percentages of HCCS (Fig. 4, 5). At the district level, the density of the insured population is classified by the district percentage of the insured population from the total insured population of a state. The classification was < 20% insured population, very low; 20–39% insured population, low; 40–59% insured population, moderate; 60–79% insured population, high; and 80–100%, very high.

**Fig. 6:**
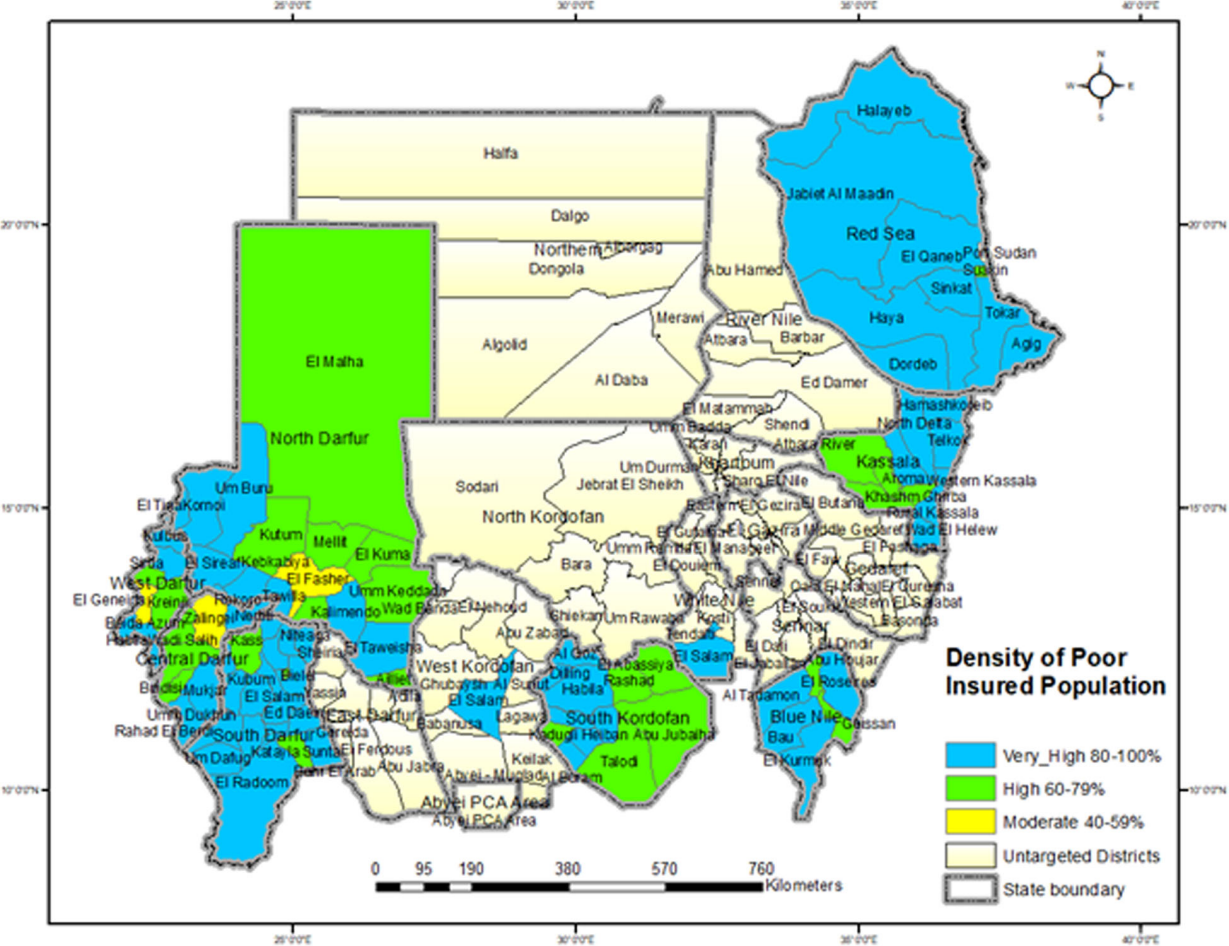
Map of Sudan, the Density of Poor Insured Population of Districts. Source: The software of the map from OCHA

In the district state analysis, the distribution of the income-insured population districts showed that the insured poor populations had high densities in district and zero catchment areas (Fig. 6). The insured rich populations had low densities in district and zero catchment areas (Fig. 6).

At the district level, the density of the insured poor population was classified by the district percentage of the insured poor population from the total insured population of a state. The classification was < 20%, 20–39%, 40–59%, 60–79%, and 80–100%, indicating very low, low, moderate, high, and very high, respectively.

## Discussion

The innovative DDADD framework in this study monitored the concept of leaving no one behind in the NHIF of Sudan and tracked the equitable implications of the mobilization of health care resources towards health equity in the SDGs. In addition, the framework detected who was being left behind, why they were being left behind, where they were being left behind and how to protect them from being left behind.

The first analytic methods of the DDADD framework identified the effect of the density of the insured population on left-behind districts’ HCC coverage. The results showed that the NHIF provided an HCC for every group of neighborhoods in high-density insured population districts in N-catchment area districts, which accounted for one-fifth of all Sudanese districts. In the very low-density insured population districts, HCCs were mobilized by providing an HCC for each district or zero. Thus, the mobilization of resources based on the density of the insured population left two-fifths of all Sudanese districts behind.

The second analytic methods of the DDADD framework explored the effect of the catchment area distribution of HCCs on insured accessibility. The results showed that district, state and national inequitable accessibility to HCCs was based on the insured population’s access to HCCs by neighborhood, subdistrict, district and zero, and this distribution left four-fifths of the insured poor behind in inequitable accessibility. The third analytic methods of the DDADD framework tracked the effect of income-insured accessibility on the income-insured utilization of health care services. The measurements showed that the insured accessed by N-catchment areas utilized more than those accessed by MTN-catchment areas, and the utilization of the insured poor accessed by N-catchment areas was higher than that of the insured poor accessed by MTN-catchment areas and the rich insured accessed by N-catchment areas. However, the insured poor accessed by MTN-catchment areas utilized less than the rich insured accessed by MTN-catchment areas. The fourth analytic methods of the DDADD framework determined the catchment area cost of health care services. The figures show that the cost of health care services increased gradually from high-density insured populations to low-density insured populations; therefore, the cost of health equity requires additional financial resources. Constructing the framework around the catchment areas of HCCs made the framework able to monitor the concept of ‘leaving no one behind’.

The results showed that the Andersen behavioral model is valuable for assessing the association of health care service utilization with the enabling factors of income insured, availability of health care services, and distance for access to health care services. These enabling factors are significant predictors of health service utilization.

In this study, unexpected findings emerged in the statistics. Khartoum State had access by N-catchment and West Kordofan State by subdistrict catchment, but the state geographical utilization rate presented a trend of a low insured utilization rate in Khartoum State and West Kordofan. Khartoum State is the capital state of Sudan, so copayment may be the factor influencing the utilization rate. The quality of health care services affected utilization in West Kordofan State because this state suffers from poor infrastructure. The quality of health care services could have a greater impact on the reduction of the rich MTN-catchment area utilization rate compared with the poor MTN-catchment area utilization rate in North Kordofan and Sinnar States. The poor N-catchment area utilization rate was lower than the rich N-catchment in River Nile State, which might be explained by copayment, which has negative effects on the poor.

The existing measurements of accessibility and health care services utilization include raster and network data models [25], Digiroad [26], the combination of land cover (GlobCover), elevation, road and river layers in AccessMod [27], Euclidian distance [28], the combination of the two-step floating catchment area method, the distance factor, the Huff-based competitive model [29], model-based estimates of average trip length [30], geographic information system-shortened path analysis and two-step floating catchment area methods [31], the index of commercial geospatial data quality/availability [32], the potential geographic accessibility in different scenarios [33], geographic information system maps [34], demographic mapping methods [35], straight-line distance, network distance, network travel time, raster travel time, mechanized, and nonmechanized [36], the two-step floating catchment area [37], and proximity [38]. These methods measure the accessibility or utilization of health care services by travel time, travel distance, travel behavior information, care resources, commercial geocoding, and neighborhood characterization. Furthermore, they measure accessibility according to time or distance traveled from the allocated HCC, so they measure the effect of accessibility on utilization.

In World Health Statistics 2018: monitoring health for SDGs [39], health equity is measured by the SDG index of essential services coverage; this compares people who have access to a full range of essential services to the average coverage [39]. It disaggregates the data according to levels of wealth and education, geographical locations within a country, age and sex [39]. This WHO framework provides national information about equity regarding the utilization of essential health services between average coverage and full coverage and between rich and poor [39]. Research studies disaggregate the data based on gender [40, 41], socioeconomic status [42–46], income [47–49], marital status [50], residence [51], ethnicity [52], and education [53, 54]. This disaggregation facilitates the detection of inequity in health between advantaged and disadvantaged groups.

The existing health equity methods aim to provide information to diagnose the equity effect of the accessibility and utilization of health care services along with the WHO’s methods of disaggregating health care services according to residence to investigate health equity between areas and between poor and rich populations. However, the methods do not connect the distribution of HCCs to accessibility to explore the poor and where people are left behind, and the methods were not formulated to provide information about underlying causes to treat inequity in accessibility. Previous studies diagnosed the gap between the poor and the rich without considering the gap within the poor in financial protection systems to discover the effect of accessibility on the insured poor’s utilization of health care services.

This research contributes to public health novel DDADD framework to monitor the concept of ‘leaving no one behind’, to track the progress in accomplishing SDGs 2030 by UHC programs. The framework disaggregates disadvantaged and advantaged groups by DDA to monitor health equity within and across the groups, to determine who is being left behind. It measures the distribution of health care resources between areas by DDA catchment areas, to determine where disadvantaged groups are being left behind. It interconnects access to health care services to the catchment areas of health care services, utilization of health care services to access, the cost of health care services to density of population coverage, in order to conceptualize the implications of resource allocation, to generate knowledge for how to reallocate resources, and to formulate policies to ensure that no one will be left behind.

The DDADD framework generates novel knowledge that fills the knowledge gap in public health to monitor the concept of ‘leaving no one behind’, to reallocate health resources towards the reduction of health inequity by UHC programs. The framework determines the equitable distribution of health care services in high-density insured population districts that protects insured poor, but the inequitable distribution in low-density insured population districts that left insured poor behind. It discovers that a majority of the population living in low-density population districts is poor and insured poor, the low-density population districts suffer lack of health care facilities, and the cost of health care services is high.

As a result, the health insurance schemes need to generate revenue to reduce health inequity to protect insured poor in low-density population districts. The premium equation of health insurance scheme is based on the probability of illness. The equation needs restructuring according to equity and the probability of illness, in order to protect insured poor in low-density population districts. Therefore, the insured population live in high-density population districts subsidize for who live in low-density population districts. The supply-side of UHC needs to invest in health care facilities in low-density population districts based on needs and equity, to accomplish UHC, and to reduce health inequity.

### Limitations

This study did not differentiate between insured areas covered or utilized by N-catchment areas within 5 km and outside of the 5 km area. It accounted for insured accessed or utilized by N-catchment areas with insured subdistrict or district catchment areas because the data were collected by district, where the unit of analysis was the district and not the HCC. This study did not identify insured children under five, insured elderly, or insured by gender with regard to being left behind in health equity, which is important for reducing the morbidity rate and the mortality rate of the targeted population.

There is an urgent need to study the health insurance schemes reliance on equity and the probability of illness, since equity will play a more pivotal role in remobilization of health resources towards the low-density population districts left behind to protect insured poor. Future studies are needed to investigate catchment area interaction mechanisms by N-catchment area within 5 km, N-catchment area outside 5 km, subdistrict, district, and zero to track insured children under five, insured elderly, and insured by gender with regard to being left behind in health equity to monitor the effects of payment mechanisms and copayment on health equity in SDGs to achieve UHC. Finally, the question of whether the quality of health care services is equitable between districts needs to be answered by another study.

## Conclusion

This study innovated the DDADD framework based on disaggregation of data around the DDA catchment areas of HCCs and by using the concept of ‘leaving no one behind’ in health equity in the SDGs. The DDADD framework has the ability to track the concept of leaving no one behind to monitor the equitable implications of the mobilization of health care resources by the NHIF towards UHC. Furthermore, it provides district, state, and national information about health equity within insured poor groups to determine who is being left behind and why, the districts that are left behind, the cost of health care services between the districts and the effect of the density of the insured population on left-behind districts. The unique characteristic of the DDADD framework is that it can consider the main function of the NHIF, which is to reallocate health care resources towards left-behind districts. The framework generated novel knowledge to public health by concluding that the equitable distribution of health care services in high-density insured population districts protects insured poor, but the inequitable distribution in low-density insured population districts leaves insured poor behind. It determines that a majority of the population living in low-density population districts is poor and insured poor, the low-density population districts suffer lack of health care facilities, and the cost of health care services is high. The policymakers in the supply-side UHC need to distribute health care services based on needs and equity in order to protect poor in low-density population districts. Health insurance schemes also require restructuring of the premium formula according to equity and the probability of illness in order to finance health care services in low-density population districts left behind UHC, to protect insured poor.

## Data Availability

The data is available.

## Abbreviations

NHIF: National Health Insurance Fund
SDGs: Sustainable Development Goals
SPSS: Statistical Package for Social Sciences
WHO: World Health Organization
UHC: Universal Health Coverage
km: kilometer
